# Cold Conditioned: Discovery of Novel Alleles for Low-Temperature Tolerance in the Vavilov Barley Collection

**DOI:** 10.3389/fpls.2021.800284

**Published:** 2021-12-15

**Authors:** Ahmad H. Sallam, Kevin P. Smith, Gongshe Hu, Jamie Sherman, Peter Stephen Baenziger, Jochum Wiersma, Carl Duley, Eric J. Stockinger, Mark E. Sorrells, Tamas Szinyei, Igor G. Loskutov, Olga N. Kovaleva, Jed Eberly, Brian J. Steffenson

**Affiliations:** ^1^Department of Plant Pathology, University of Minnesota, St. Paul, MN, United States; ^2^Department of Agronomy and Plant Genetics, University of Minnesota, St. Paul, MN, United States; ^3^USDA-ARS, Small Grains and Potato Germplasm Research, Aberdeen, ID, United States; ^4^Department of Plant Sciences and Plant Pathology, Montana State University, Bozeman, MT, United States; ^5^Department of Agronomy and Horticulture, University of Nebraska-Lincoln, Lincoln, NE, United States; ^6^University of Wisconsin and UW-Extension, Alma, WI, United States; ^7^Department of Horticulture and Crop Science, The Ohio State University/Ohio Agricultural Research and Development Center (OARDC), Wooster, OH, United States; ^8^Department of Plant Breeding and Genetics, Cornell University, Ithaca, NY, United States; ^9^N.I. Vavilov Institute of Plant Genetic Resources (VIR), Saint Petersburg, Russia; ^10^Central Agricultural Research Center, Montana State University, Moccasin, MT, United States

**Keywords:** barley, low-temperature tolerance, winter survival, germplasm resources, genomewide association study, facultative growth habit

## Abstract

Climate changes leading to higher summer temperatures can adversely affect cool season crops like spring barley. In the Upper Midwest region of the United States, one option for escaping this stress factor is to plant winter or facultative type cultivars in the autumn and then harvest in early summer before the onset of high-temperature stress. However, the major challenge in breeding such cultivars is incorporating sufficient winter hardiness to survive the extremely low temperatures that commonly occur in this production region. To broaden the genetic base for winter hardiness in the University of Minnesota breeding program, 2,214 accessions from the N. I. Vavilov Institute of Plant Industry (VIR) were evaluated for winter survival (WS) in St. Paul, Minnesota. From this field trial, 267 (>12%) accessions survived [designated as the VIR-low-temperature tolerant (LTT) panel] and were subsequently evaluated for WS across six northern and central Great Plains states. The VIR-LTT panel was genotyped with the Illumina 9K SNP chip, and then a genome-wide association study was performed on seven WS datasets. Twelve significant associations for WS were identified, including the previously reported frost resistance gene *FR-H2* as well as several novel ones. Multi-allelic haplotype analysis revealed the most favorable alleles for WS in the VIR-LTT panel as well as another recently studied panel (CAP-LTT). Seventy-eight accessions from the VIR-LTT panel exhibited a high and consistent level of WS and select ones are being used in winter barley breeding programs in the United States and in a multiparent population.

## Introduction

The Upper Midwest was once the largest malting barley (*Hordeum vulgare*) production region in the United States, supplying the main raw ingredient for many large and small breweries throughout the country. Over the past few decades, barley hectarage in the region has dropped to historic lows from 3,44,000 ha in 1990 to less than 38,000 ha starting in 2010 due to competition from crops such as corn (Zea mays) and soybeans (Glycine max), the fallout from the devastating Fusarium head blight epidemics that started in the early 1990s, and the stagnation of grain yield gains in barley in comparison with other crops (USDA National Agricultural Statistics Service (NASS); Windels, 2000; Bootsma et al., 2005; USDA NASS, 2020). Despite this downward trend, there is optimism for a recovery of some production due to several factors, including the dramatic upsurge of craft breweries creating a demand for locally produced malting barley, increasing ecological pressures on the predominant corn/soybean cropping system that threatens its sustainability, and adaptation of autumn-sown cover crops in production systems that could evolve to winter annuals, including winter barley.

Barley is a cool season annual crop and has been widely cultivated in the Upper Midwest for about 150 years (Weaver, 1943). However, recent climatic changes have led to markedly higher summer temperatures (USGCRP, 2017) that can adversely affect the productivity of spring barley (Savin and Nicolas, 1996; Mahalingam and Bregitzer, 2019). One option for escaping the adverse effects of elevated summer temperatures and drought conditions is to develop winter or facultative growth habit cultivars that can be sown in autumn (mid-September to early October) and harvested in early summer (late June-early July). Such cultivars can also accommodate a double cropping scheme with short season pea (Pisum sativum) and soybean cultivars being planted immediately after the barley harvest. While autumn-sown barley with either a winter or facultative growth habit has been a reliable crop for states in the central and southern Great Plains, Mid-Atlantic, and Pacific Northwest regions of the United States, it is not considered such in the Upper Midwest due to extremely low winter temperatures. Still, given the increasing frequency of mild winters in the Upper Midwest together with a concerted breeding effort for low-temperature tolerance (LTT), it is possible that autumn-sown barley could become a viable crop option for producers. In this report, we will use LTT and winter survival (WS) interchangeably as the former is the major component of the latter.

Autumn-sown barley has many advantages over the traditional spring-sown barley cropping system that has historically been used in the Upper Midwest, including higher yield potential, earlier harvest, enhancement of some malting quality parameters, higher nitrogen and water use efficiency, and escape from some major “summer” diseases (Ellis and Russell, 1984; Pržulj et al., 1998; Nielsen and Munck, 2003; Zhong et al., 2019). Ellis and Russell (1984) conducted field trials to compare the agronomic traits of winter vs. spring barleys under autumn and spring sowing. All winter and spring barleys had a higher number of spikes and grain yield under autumn sowing compared with spring sowing. In addition to agronomic traits, winter growth habit cultivars can also produce higher amounts of malt extract with less protein content than spring cultivars; however, they did contain higher levels of β-glucan that can reduce malting quality (Pržulj et al., 1998; Nielsen and Munck, 2003).

Breeding for LTT in small grain cereals is challenging due to the lack of effective screening protocols, the limited genetic diversity for the trait, and the difficulty of combining favorable agronomic and quality alleles with LTT (Fowler and Gusta, 1979). A program focused on breeding autumn-sown barley cultivars for the Upper Midwest region was started in 2009 at the University of Minnesota (UM). The highest priority trait for this program is LTT because without it there would be no surviving crop in most years. Barley is often classified on the basis of its growth habit into three types: winter, spring, and facultative. Winter barley requires vernalization (i.e., cold period) to flower and is therefore sown in the autumn to satisfy this condition. However, to survive through this cold period, winter barleys must have some LTT, the level of which depends on the severity of winter in the target environment. Spring barleys do not require vernalization, are sown in the spring, and possess no appreciable level of LTT. Although facultative types do not require vernalization, some lines possess levels of LTT comparable to winter barley (von Zitzewitz et al., 2005; Rizza et al., 2011). This makes facultative types more flexible for cropping systems because they can be sown either in the spring or autumn. Additionally, facultative types facilitate breeding because they do not require an extended cold treatment to initiate vernalization and thus have a much shorter seed-to-seed generation time which translates into shorter breeding cycles.

WS, as assessed in field trials, is the most robust test for a genotype’s winter hardiness. This is because WS encompasses all factors affecting the ability of plants to persist to the following spring through the early growth and cold hardening periods in autumn, the long duration of low-temperature stress in winter, and extreme temperature fluctuations and freeze/thaw cycles in the spring (Galiba et al., 2009; Zhong et al., 2019). Depending on the weather pattern (chiefly maximum low temperature and duration as well as snow levels, which can insulate the crop) in any given year, field evaluations of autumn-sown barley germplasm can sometimes result in either complete winter kill or complete winter survival (Gusta et al., 1982). Therefore, WS trials established at sites with variable cold stress levels can provide valuable information on the response of different barley genotypes. Winter hardy cultivars of small grain cereal crops have tolerance to low temperatures, the duration of exposure to these low temperatures, flooding, and ice incasement (Gusta et al., 1982; Galiba et al., 2009). WS of species in the *Triticeae* is a complex trait that prominently includes tolerance to sub-freezing temperatures (commonly referred to as LTT), vernalization sensitivity, and photoperiod sensitivity (Hayes et al., 1993).

In temperate cereal crops, LTT is under the control of frost resistance (*FR*) genes. The frost resistance gene *FR1* was mapped on the long arm of chromosome 5A in wheat (*Triticum aestivum*), tightly linked to the vernalization sensitivity gene *Vrn1* and was suggested to be syntenic to the *FR-H1* gene in barley (Hayes et al., 1993; Galiba et al., 1995). In barley, three *FR* genes (*FR-H1*, *FR-H2*, and *FR-H3*) were identified. *FR-H1* was first reported in the Dicktoo/Morex double haploid mapping population and was mapped on chromosome 5H (Hayes et al., 1993). *FR-H1* co-segregated with the barley vernalization sensitivity gene *Vrn-H1* on the long arm of chromosome 5H (Francia et al., 2004). Later, *FR-H1* and *FR-H2* were mapped and found to be approximately 34 cm apart with *FR-H1* lying distal to *FR-H2* (Francia et al., 2007). The frost resistance gene *FR-H3* was identified on the short arm of chromosome 1H in two doubled haploid mapping populations and explained about 48% of the variability for LTT (Fisk et al., 2013). In addition to the frost resistance genes, vernalization sensitivity genes also contribute to winter hardiness. A two-locus epistatic model was proposed for vernalization sensitivity in barley. Vernalization downregulates *Vrn-H2* and upregulates *Vrn-H1*, which promotes flowering (Yan et al., 2003). *Vrn-H2* was mapped on the long arm of chromosome 4H and encodes a zinc finger-CCT domain (ZCCT) that represses flowering. *HvBM5A* is the candidate gene for *Vrn-H1* and is located on chromosome 5H. This gene encodes a MAD-box floral meristem transcription factor that is upregulated under vernalization and long day conditions (Yan et al., 2003; Trevaskis et al., 2007). Deletion of the *Vrn-H2* candidate gene resulted in the lack of vernalization requirement in facultative and spring barleys from North America and Europe (von Zitzewitz et al., 2005). An important point to note is that vernalization itself is not required for winter hardiness. In barley genotypes with a winter growth habit, vernalization provides a mechanism for preventing the transition from the vegetative phase to flowering when the weather is too cold, thereby avoiding damage to the plant. For barley genotypes with a facultative growth habit, such damage can be avoided by having a long-day photoperiod requirement to initiate flowering and of course an adequate level of LTT.

Several genome-wide association studies (GWAS) have been conducted to map LTT genes in barley using diverse germplasm panels (von Zitzewitz et al., 2011; Visioni et al., 2013; Muñoz-Amatriaín et al., 2020). Although several genes and quantitative trait loci (QTL) for LTT were identified in these investigations, it is not known whether they would confer sufficient winter hardiness for barley to survive the typically severe winters in the Upper Midwest region. To identify additional sources of LTT, we evaluated a panel of barley accessions (*N* = 2,214) from the N. I. Vavilov Institute of Plant Industry (abbreviated VIR in Russian) in St. Petersburg, Russia. While the VIR collection contained a diverse array of landraces, cultivars, and breeding lines from around the world, it also included a concentration of unique germplasm from the Russian Federation and former U.S.S.R. In the autumn of 2014, the VIR panel was sown at the Minnesota Agricultural Experiment Station in St. Paul and assessed for survival in the spring of 2015. Surprisingly, 267 (12%) accessions survived the harsh winter of 2014–15 (ambient low temperatures of −30°C), when in a parallel trial over 99% of lines from the UM barley breeding program died. These survivors (hereafter designated the VIR-LTT panel) represent a possible new and diverse gene pool to breed barley for enhanced LTT. Thus, the objectives of this research were: (1) to characterize the VIR-LTT panel for LTT across years and different locations in the United States; (2) assess vernalization sensitivity under Minnesota conditions; (3) identify genetic variants associated with LTT in the VIR-LTT panel; (4) define favorable haplotype alleles at significant associations from both the VIR-LTT panel and also the CAP-LTT panel recently reported by Muñoz-Amatriaín et al. (2020); and (5) investigate the effect of selection for LTT in the VIR-LTT panel to identify accessions with the highest level of winter hardiness.

## Materials and Methods

### Plant Materials

Accessions in the VIR-LTT panel originated from 35 countries with the largest number coming from Russia (108) followed by Ukraine (32) and Bulgaria (23) (Supplementary Table S1). Five barley controls with different growth habits and winter hardiness levels were also included in each trial: Stander (PI 564743; spring habit with no appreciable LTT), McGregor (winter habit with moderate LTT), Dicktoo (CIho 5529; facultative habit with moderate LTT), Charles (PI 637845; winter habit with moderate LTT), and Maja (winter habit with moderate LTT). Winter wheat (Norstar, CItr 17735; winter habit with high LTT; Grant, 1980) and winter rye (*Secale cereale*; Rymin, CIse 176, winter habit with high LTT; Robinson, 1973) were also included in the trials for comparative purposes with other small grain cereal crops known to have higher levels of winter hardiness than barley under Upper Midwest conditions.

### Phenotyping for LTT

After the initial winter hardiness trial of VIR germplasm in 2014–15 at St. Paul, Minnesota (N 44° 59' 51.22'', W 93° 10' 26.19''; abbreviated as SP14.15), the VIR-LTT panel was evaluated 10 additional times at the following sites and years: Crookston, Minnesota (N 47° 46' 26.39'', W 96° 36' 29.22'') in 2015–2016 (CK15.16), St. Paul in 2015–2016 (SP15.16), Crookston in 2016–2017 (CK16.17), St. Paul in 2016–2017 (SP16.17), Aberdeen, Idaho (N 42° 56' 38.68'', W 112° 50' 17.98'') in 2016–2017 (AB16.17), Bozeman, Montana (N 45° 40' 45.95'', W 111° 02'38.57'') in 2016–2017 (BZ16.17), Mead, Nebraska (N 41° 13' 35.54'', W 96° 29' 21.33'') in 2016–2017 (ME16.17), Alma, Wisconsin (N 44° 19' 11.88'', W 91° 54' 53.58'') in 2016–2017 (AL16.17), Ithaca, New York (N 42° 26' 22.57'', W 76° 29' 48.49'') in 2016–2017 (IT16.17), and Rosemount, Minnesota (N 44° 44' 21.07'', W 93° 07' 33.99'') in 2019–2020 (RS19.20). The five barley controls along with Norstar wheat and Rymin rye were included with four replicates in each trial. Accessions were sown in the autumn according to the usual planting times and husbandry practices for each site. In all WS experiments, 4 g of seeds were machine planted into a single row plot 1.0 m in length. Stand count assessments (0–100% in 10% increments) were made in the autumn prior to freeze-up at most sites. In the spring, WS was assessed as the percentage of plants surviving the winter on a 0–100% scale in 10% increments. To determine vernalization sensitivity, seeds of the VIR-LTT panel were directly planted in the field as hill plots (25 seeds/hill) in the spring of 2016 at St. Paul. Observations were then taken for growth habit and heading date. Accessions that did not head under the long-day environment of the Minnesota summer were considered true winter types requiring vernalization. Those that did head by the end of July were considered facultative types that did not require vernalization. Moreover, all accessions were scored for days to heading at SP16.17, which was expressed as the number of days from planting in the fall to when 50% of spikes within a plot emerged from the flag leaf sheath (boot) in the spring. To determine the effectiveness of phenotypic selection for WS, we imposed selection on the VIR-LTT panel. A 78 accession subset of the VIR-LTT panel (hereafter referred to as VIR-LTT78) exhibiting the highest average WS and also the greatest WS consistency (>50%) across trials was evaluated further (four additional trials) in Moccasin, Montana (N 47° 03' 49.82'', W 109° 57' 43.80'') in 2017–2018 (MC17.18), Mead in 2017–2018 (ME17.18), Ithaca in 2017–2018 (IT17.18), and St. Paul in 2020–2021 (SP20.21).

### Genotyping

All accessions of the VIR-LTT panel were grown in the greenhouse to the three-leaf stage. Then, the second leaves were cut-off from the plants using a scissors and inserted into the wells of a 96-deep-well plate to which silica gel was added to desiccate the tissue. The desiccated leaf samples were then sent to the USDA-ARS North Central Small Grains Genotyping Laboratory in Fargo, North Dakota, for genotyping. Accessions were genotyped with the Barley 9K iSelect array (Comadran et al., 2012). Allele calls were scored using Genome Studio v2011.1 (Cavanagh et al., 2013). The Genome Studio module identified different clusters that represent AA, AB, and BB genotypes in biallelic markers. Clustering for all markers was visualized to determine the accuracy of allele calling. Markers were filtered to exclude those with a minimum allele frequency (MAF)<0.01. The barley genome assembly (Mascher et al., 2017) was used to obtain the physical positions for all SNP markers that passed filtering in the VIR-LTT panel. Genotypic data for the VIR-LTT panel are included in Supplementary Table S2.

### Data Analysis

#### Phenotypic Data Analysis

For WS, a mixed linear model (MLM) implemented in the MIXED procedure of SAS was used to correct for differences in the means of trials (v.9.4, SAS Institute Inc., 2013). Analysis of variance was performed for WS across all trials using the PROC GLM procedure in SAS. In each trial, studentized residuals were estimated for all accessions. Accessions with absolute studentized residuals of 3.0 or more were excluded and considered as missing data. Common barley controls across the VIR-LTT (for all 267 accessions) and VIR-LTT78 (for the selected 78 accessions) trials were used to correct for variance in trial means using the MIXED procedure is SAS (Sallam and Smith, 2016). Broad-sense heritability (*H*) on an entry mean basis was estimated for WS using the equation:


[1] $$H=\sigma_{g}^{2}/\left(\sigma_{g}^{2}+\sigma_{e}^{2}/n\right),$$

where $\sigma_{g}^{2}$ is genetic variance, $\sigma_{e}^{2}$ is the pooled error variance that includes G × E and residuals, and *n* is the number of trials.

#### Characterization of Linkage Disequilibrium and Assessment of Population Structure

Adjacent linkage disequilibrium (LD) was estimated as *r^2^* for all seven chromosomes in TASSEL (Bradbury et al., 2007). The distribution of adjacent marker LD was visualized using JMP (JMP, version 13.1.0, SAS Institute Inc., Cary, NC, United States). To determine LD decay in the VIR-LTT panel, a sliding window of 50 adjacent markers (LD_50_) was used to estimate LD as *r^2^* across all chromosomes. LD decay was visualized by plotting the LD_50_ and physical distance in JMP. LD estimates among markers in the significant regions were used to assess whether closely linked associations represent the same or independent loci. We used a LD of 0.2 or more for defining if two adjacent markers are in LD. Population stratification in the VIR-LTT panel was characterized using principal component analysis (PCA) as performed in TASSEL. To characterize the genetic relatedness of accessions in the VIR-LTT panel, genetic relationships were estimated as the realized additive relationship matrix in the rrBLUP package of R using all available markers (Endelman, 2011). The genomic additive relationship matrix was estimated as:


[2] $$K=\frac{ZZ^{\prime }}{2\sum p_{i}\left(1-p_{i}\right)}$$

where: *Z* = *M*−*P*. *M* is a matrix of VIR-LTT accessions by SNP loci marker containing genotype values (1, 0, −1), and *P* is a vector of markers containing allele frequencies expressed as 2 (*p_i_*–0.5) with *p_i_* representing the allele frequency of marker *i* (VanRaden, 2008). Pairwise genetic distance was calculated among all VIR-LTT accessions as 1 – identity-by-state in TASSEL. *K*-means clustering was performed on the VIR-LTT accessions using the genetic distance matrix in the Hartigan–Wong algorithm implemented in R (R Core Team, 2020). A total of 5,000 iterations were used in *K*-means clustering. Clusters identified by *K*-means clustering were displayed on the plot of the first two principal components. The PCA plot for cluster analysis was created in JMP.

Using common markers between the VIR-LTT and the CAP-LTT panels, PCA for both panels was performed as described above (see also Muñoz-Amatriaín et al., 2020). Genetic relatedness between accessions in the two panels was characterized based on the realized additive relationship matrix estimated as described above using the common markers.

#### Genome-Wide Association Study and Identification of Candidate Genes

Genome-wide association studies was performed on the VIR-LTT panel to identify genetic variants with enhanced levels of winter hardiness. A MLM was used that accounted for both population stratification (Q) and genetic relatedness (K; Yu et al., 2006). In the Q + K model, the mixed model is described as


[3] y=μ+Wm+Qv+Zu+ε

where y is the vector of the mean trial adjusted WS for each accession across trials, μ is population mean, m is the vector of fixed SNP effects, v is the vector of fixed population effect, u is the vector of random genetic background effect for each accession, and ε is the vector of residuals. W, Q, and Z are incidence matrices. The parameter u is distributed as $N\left(0\mathrm{,K}\sigma_{g}^{2}\right)$, where K is the kinship matrix and $\sigma_{g}^{2}$ is the genetic variance. The parameter ε is distributed as $N\left(0\mathrm{,I}\sigma_{\varepsilon }^{2}\right)$, where I is the identity matrix and $\sigma_{\varepsilon }^{2}$ is the error variance. The first two PCs and the realized additive relationship matrix were used to account for population structure and genetic relatedness among the 267 accessions in the MLM. The Q + K association mapping model was implemented in the rrBLUP package in R (Endelman, 2011). Multiple testing comparisons for GWAS were taken into account using the False Discovery Rate at a significance level of 0.05 to control for type I errors (Benjamini and Hochberg, 1995; Storey and Tibshirani, 2003). The proportion of phenotypic variance explained by markers (*R*^2^) was calculated as *R^2^* = SS_reg_/SS_tot_, where SS_reg_ is the regression sum of squares and SS_tot_ is the total sum of squares of the regression model.

For each SNP found significant in the GWAS analysis, haplotypes of three adjacent markers – including the most significant marker in the middle of the haplotype – were used for gene annotation. The gene annotation information for the barley reference genome is available at the IPK Barley BLAST Server.^1^ Confirmation of the BLAST search was performed in BARLEX (Colmsee et al., 2015), and the names of candidate genes located within 1 Mbp from the haplotypes were reported.

We compared the GWAS results for WS in the VIR-LTT panel with those recently described for the CAP-LTT panel (Muñoz-Amatriaín et al., 2020) to identify QTLs for the trait that were unique to each germplasm panel or common to both. The only barley accessions common to both panels were Charles, Dicktoo, and McGregor (used only as controls in the VIR-LTT panel), which were used to adjust for trial means using the MIXED procedure in SAS (Sallam and Smith, 2016). Both panels were genotyped with Barley 9K iSelect array, enabling comparison of estimated haplotype frequencies and scores. For this comparative analysis, we obtained all raw genotypic and phenotypic data for the CAP-LTT panel from the investigators who conducted the study. In both the VIR-LTT and CAP-LTT panels, we generated haplotypes of three adjacent markers – as described above – in the genomic regions associated with WS (Muñoz-Amatriaín et al., 2020). In both panels, haplotype parameters and associations with WS were estimated using the haplo.stats package in R (Schaid et al., 2002). To correct for multiple testing, we used simulate = TRUE in the haplo.score function in the R package to obtain simulated values of *p*. In haplo.stats, the expectation–maximization algorithm was used to calculate haplotype frequencies followed by the estimation of haplotype scores in both panels.

To examine the effect of selection in altering the frequency of favorable alleles for WS, we evaluated the changes in the most favorable haplotype allele frequencies at all 12 significant Marker Trait Associations (MTAs) between the 267 accessions in the VIR-LTT panel and the 78 accessions in VIR-LTT78 selection panel. A paired *t test* was used to assess the statistical significance for changes in the frequency of the most favorable haplotype alleles between the VIR-LTT panel and VIR-LTT78 subset.

## Results

### Winter Survival in the VIR-LTT Panel

**Figure 1:**
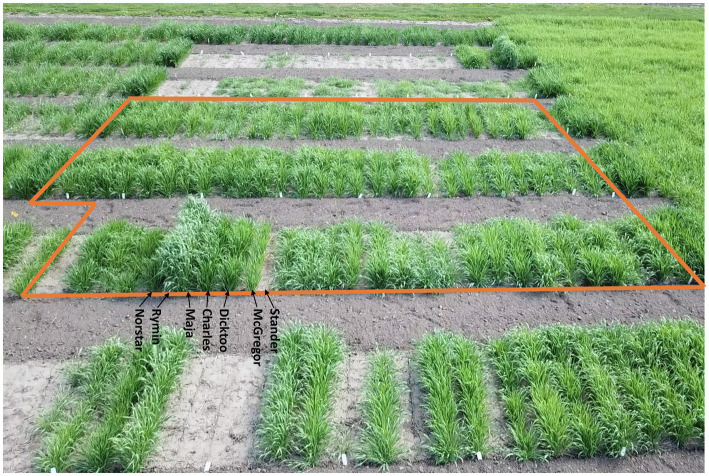
View of the winter hardiness trial planted on the St. Paul campus of the University of Minnesota in 2020–21. The VIR-LTT78 panel is demarcated by the orange line with most accessions exhibiting a very high level of winter survival. In the foreground, the standard control lines of Norstar (winter type wheat), Rymin (winter type rye), Maja (winter type barley), Charles (winter type barley), Dicktoo (facultative type barley), and McGregor (winter type barley) also exhibited a high level of winter survival, whereas Stander (spring type barley) died out completely.

**Figure 2:**
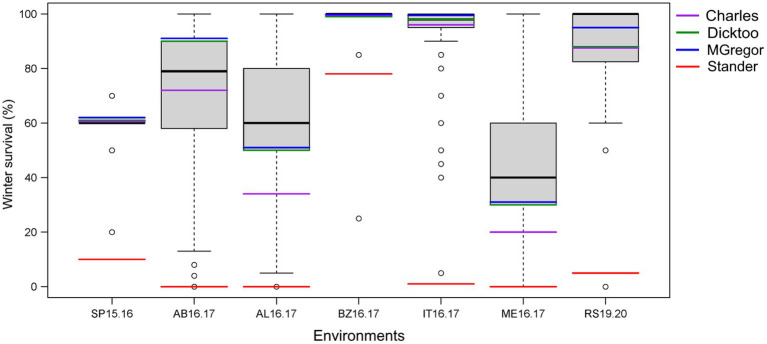
Phenotypic distribution of average winter survival of the 267 barley accessions of the VIR-LTT panel grown in seven trials in six US states in comparison to the barley controls of Stander (spring growth habit), McGregor (winter habit), Dicktoo (facultative habit), and Charles (winter habit). The environments are St. Paul, MN 2015–2016 (SP15.16); Aberdeen, ID 2016–2017 (AB16.17); Alma, WI 2016.2017 (AL16.17); Bozeman, MT 2016–2017 (BZ16.17); Ithaca, NY 2016.2017 (IT16.17); Mead, NE 2016.2017 (ME16.17); and Rosemount, MN 2019–2020 (RS19.20).

Line names and other identifiers for the 267 VIR-LTT accessions are given in Supplementary Table S1, along with their passport information, improvement history, cluster analysis assignment, WS across the 10 trials, heading date, and growth habit. Of the 267 accessions, 116 are landraces, 132 are cultivars, and 19 have an unknown improvement history. WS for the controls varied greatly across the 10 environments. The harshest winter environments occurred in the most northern site of Crookston (CK15.16 and CK16.17) where all barley controls and VIR-LTT accessions died off completely. Norstar wheat and Rymin rye had a very high rate of WS of at least 90% at Crookston. In SP16.17, the barley controls of Stander, Charles, Dicktoo, and Maja died off completely, while Norstar wheat and Rymin rye survived at 90%. As expected, the spring habit barley control of Stander died off completely in all three of these Minnesota environments. In SP16.17, only four accessions (VIR22707, VIR13651, VIR13644, and VIR13168) survived at 50% or higher. Because no or very few accessions survived at CK15.16, CK16.17, and SP16.17, these data were not included in the final analysis. For the final analysis of WS in the VIR-LTT panel, seven environments were included: SP15.16, AB16.17, AL16.17, BZ16.17, IT16.17, ME16.17, and RS19.20 (Table 1). Across these seven environments, Norstar wheat and Rymin rye showed the highest WS of 94.3 and 90.8%, respectively (Table 1). Stander, the spring habit barley control, died out completely in AB16.17, AL16.17, IT16.17, and ME16.17 and showed very low survival in SP15.16 and RS19.20 (Table 1; Figure 1). The only location where Stander survived at a high percentage (77.5%) was in BZ16.17. Thus, in all locations except BZ16.17, it was possible to eliminate most accessions with no or just a modicum of LTT. Overall, Stander had the lowest WS among the barley controls at 13.2%. In contrast, McGregor had the highest WS at 75.7% followed by Dicktoo (73.8%), Charles (67.0%), and Maja (64.7%; Table 1; Figure 1). The overall average survival for the VIR-LTT panel varied greatly from 44.8% at ME16.17 to 99.7% at BZ16.17 (Table 1). The distribution for WS varied among the seven environments with narrow distributions and moderately high to high survival rates observed at SP15.16, BZ16.17, and IT16.17 (Figure 2). WS distribution at IT16.17 was greater than at SP15.16 and BZ16.17, revealing differences in LTT among the accessions in the panel while also showing complete winter kill of Stander (Figure 2). Wide WS distributions were observed at AB16.17, AL16.17, ME16.17, and RS19.20 with all environments having very low survival rates of Stander. ANOVA showed significant environmental and genetic effects for WS in the VIR-LTT panel (Table 2). The estimation of broad-sense heritability was 0.46 for WS across all seven trials.

**Table 1:** Average winter survival (WS; %) of 267 Vavilov Institute of Plant Industry (VIR) accessions in seven field trials across six US states in comparison with barley (Stander, Charles, Dicktoo, Maja, and McGregor), wheat (Norstar), and rye (Rymin) controls.

Environments*^a^*	VIR-LTT	Stander	Charles	Dicktoo	Maja	McGregor	Norstar	Rymin
SP15.16	59.9	10.0	60.0	61.0	30.0	62.0	100.0	-
AB16.17	71.9	0.0	71.8	90.4	69.1	91.0	100.0	100.0
AL16.17	61.2	0.0	33.8	50.0	25.0	51.3	65.0	65.0
BZ16.17	99.7	77.5	100.0	99.0	100.0	100.0	100.0	100.0
IT16.17	93.8	0.0	95.8	98.4	94.0	99.5	100.0	100.0
ME16.17	44.8	0.0	20.0	30.0	40.0	31.3	95.0	97.5
RS19.20	91.4	5.0	87.5	87.5	95.0	95.0	100.0	82.5
Average	74.7	13.2	67.0	73.8	64.7	75.7	94.3	90.8

a Environments are St. Paul, MN 2015–2016 (SP15.16); Aberdeen, ID 2016–2017 (AB16.17); Alma, WI 2016.2017 (AL16.17); Bozeman, MT 2016–2017 (BZ16.17); Ithaca, NY 2016r2017 (IT16.17); Mead, NE 2016.2017 (ME16.17); and Rosemount, MN 2019–2020 (RS19.20).

**Table 2:** Mean squares for winter survival in the ANOVA for 267 VIR barley accessions grown in seven field trials across six US states.

Source of variation	Mean squares
Accession	399^***^
Environments	114407^***^
Residuals	214

Significance codes: 0.0001 or less “^***^”; 0.001 “^**^”; and 0.01 “^*^”.

**Figure 3:**
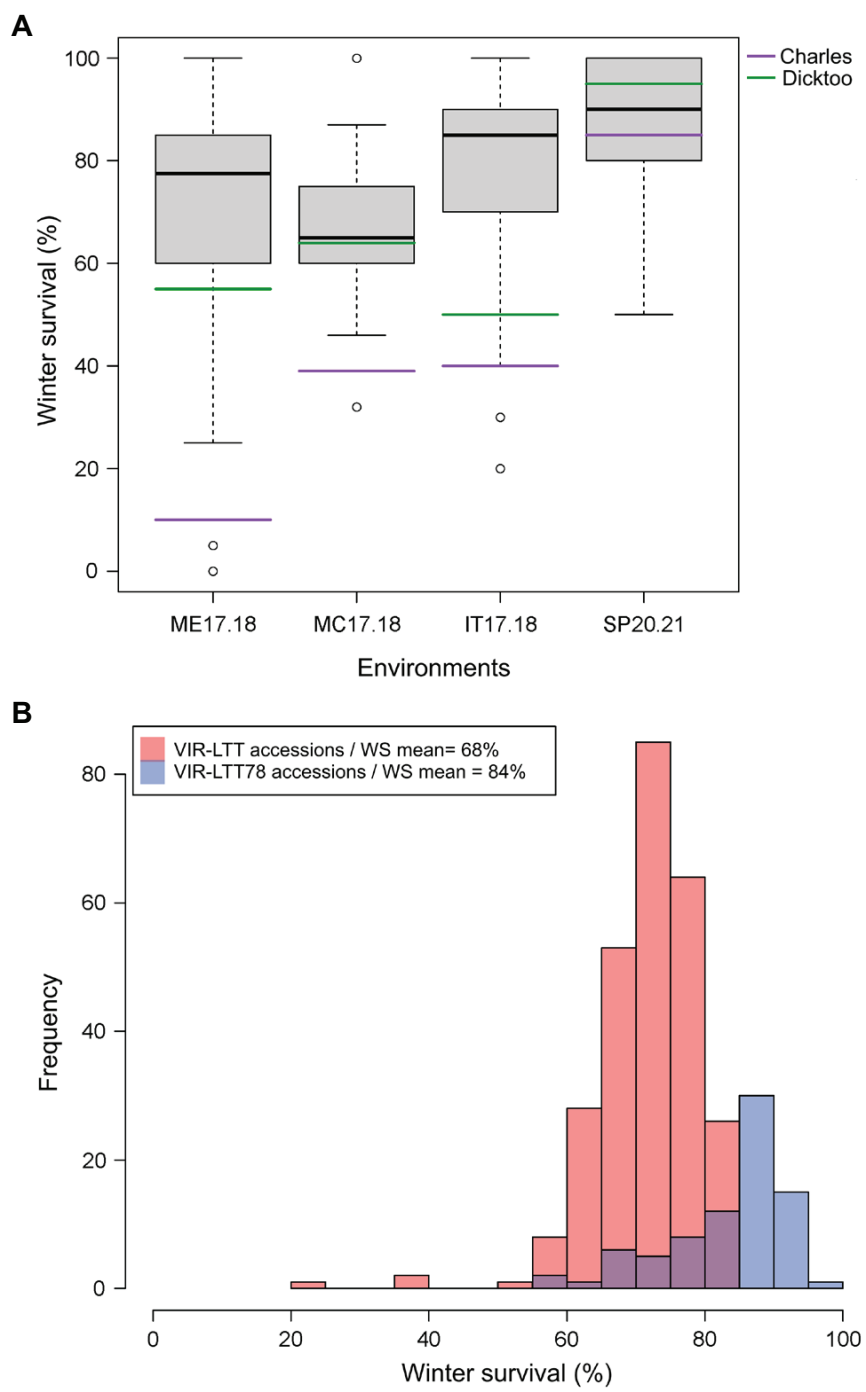
**(A)** Winter survival (WS) distribution for accessions in the VIR-LTT78 subset evaluated in Mead, NE 2017–2018 (ME17.18), Moccasin, MT 2017–2018 (MC17.18), Ithaca, NY 2017–2018 (IT17.18), and St. Paul, MN 2020–2021 (SP20.21). **(B)** Frequency of WS for the VIR-LTT panel (267 accessions evaluated in seven trials) and VIR-LTT78 subset (78 accessions evaluated in four trials) with mean of winter survival for both panels after correction of trial means using the two common barley checks of Charles (winter habit) and Dicktoo (facultative habit).

The VIR-LTT panel displayed a high overall survival rate as 264 of the 267 accessions exhibited an average survival rate of 50% or more across the seven field trials (Supplementary Table S1). The exceptions were accessions VIR17623, VIR20387, and VIR23121, which exhibited an overall WS of <50% across the seven trials because they had low survival in at least four different environments. WS was highest at BZ16.17 among all trials; however, two of the previous mentioned accessions (VIR17623 and VIR20387) had the lowest performance in this testing environment. Based on WS data from the seven environments for the VIR-LTT panel, we selected a subset of 78 accessions (designated as VIR-LTT78) that exhibited the overall highest and most consistent (50% or more) WS. The VIR-LTT78 accessions were tested in four additional environments: MC17.18, ME17.18, IT17.18, and SP20.21 (Figure 3A). In these additional test sites, two of the four barley controls (Charles and Dicktoo) were included and used to correct for differences in the trial means between the seven original environments that included the complete VIR-LTT panel and the four subsequent environments that only included the VIR-LTT78 accessions. The WS means of the entire VIR-LTT panel and the VIR-LTT78 selected accessions were 68 and 84%, respectively, after correction of trial means using the two barley controls (Figure 3B). For accessions in the VIR-LTT78 panel, we estimated the average WS across the seven trials and four additional trials to generate an average WS performance across 11 environments (Supplementary Table S3). The average survival across the 11 environments was 82.7%, which was higher than the survival of controls Charles (57.0%) and Dicktoo (76.3%; Supplementary Table S3).

To assess the vernalization requirement of the VIR-LTT panel, non-vernalized seed of each accession was directly sown in the field in April 2016 at St. Paul. Observations were then taken for early growth habit and heading date. Accessions that did not head under the long-day environment of the Minnesota summer were considered true winter types requiring vernalization. Those that did head by the end of July were considered facultative types because they did not require vernalization and were already demonstrated to possess some level of LTT. Of the 267 VIR-LTT accessions, 136 had a winter growth habit and 131 had a facultative growth habit (Supplementary Table S1).

### Marker Data, Linkage Disequilibrium, and Population Structure in the VIR-LTT Panel

**Figure 4:**
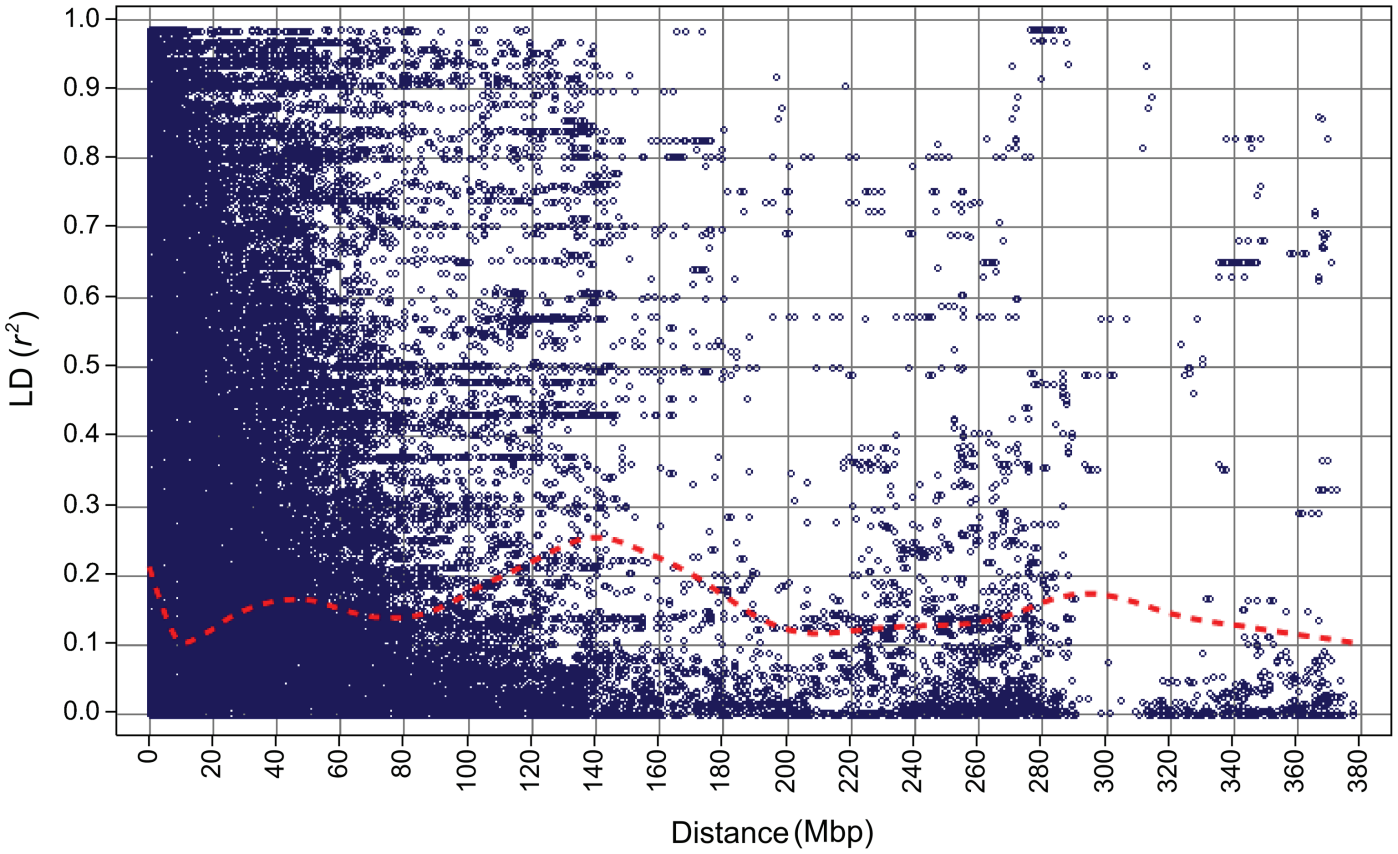
Linkage disequilibrium decay for the 267 barley accessions of the VIR-LTT panel estimated using 5,814 SNP markers.

After marker filtering, 5,813 markers with physical map positions were included in the final analysis (Supplementary Table S2). After ordering markers based on their physical map positions, the average adjacent marker LD (estimated as *r^2^*) across all chromosomes was 0.39 (LD ranged between 0.36 and 0.43; Supplementary Figure S1). LD decayed to 0.1 at about 10 Mbp, but increased again to at least 0.2 between 110 and 170 Mbp (Figure 4). LD did not decay below 0.1 for about 380 Mbp (Figure 4).

**Figure 5:**
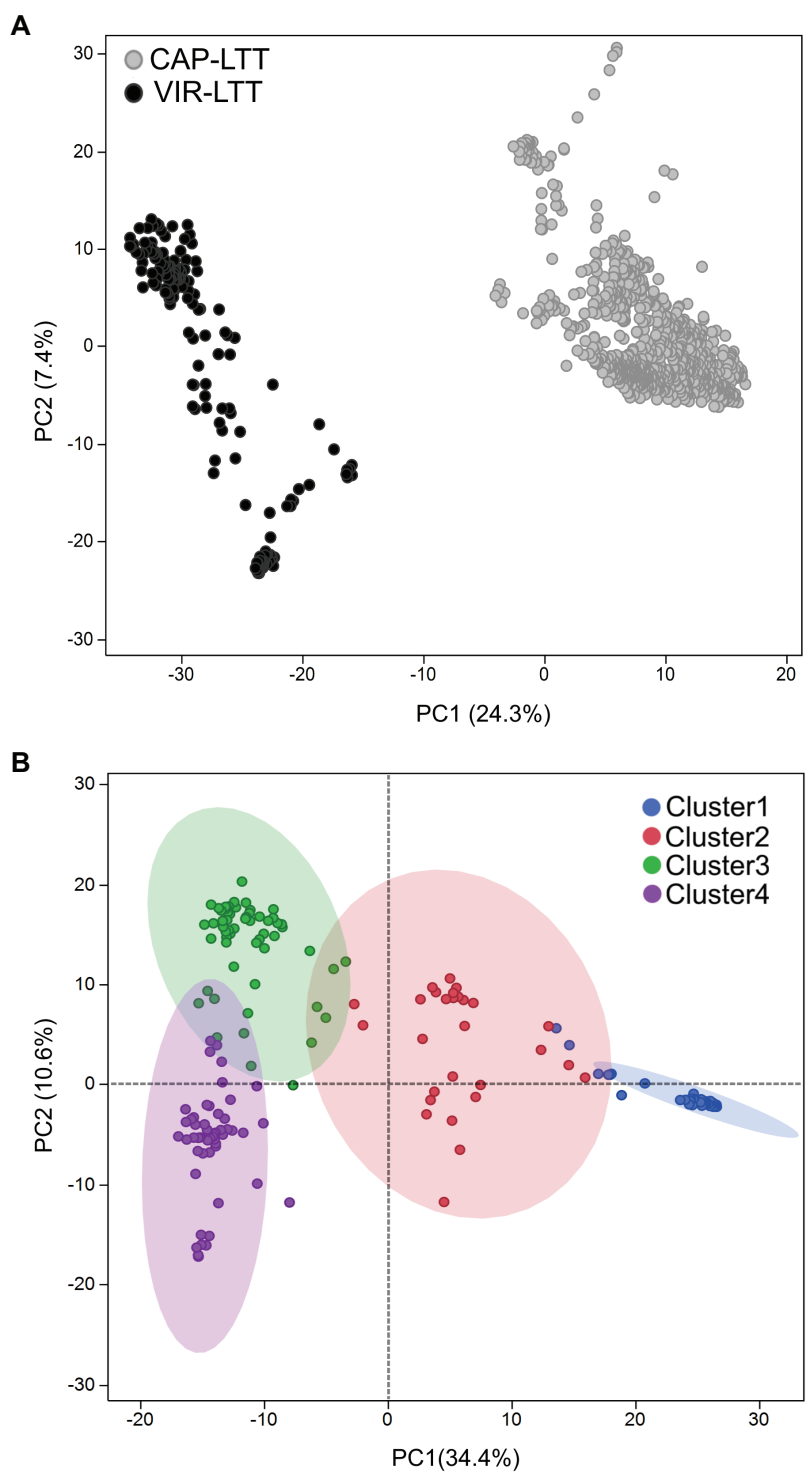
**(A)** Principal component analysis (PCA) for the 882 accessions of the CAP-LTT panel and 267 accessions of the VIR-LTT panel using 4,765 markers shared between the two panels. **(B)** Population stratification for the 267 barley accessions of the VIR-LTT panel. Four clusters were inferred from *K*-means clustering and visualized on the PCA plot.

In comparing the genotyping data between the VIR-LTT and CAP-LTT panels, we found 4,765 Barley 9K iSelect SNPs in common. These common markers were then used in PC analysis and for estimating genetic relatedness. PC analysis clearly separated the CAP-LTT panel from the VIR-LTT panel (Figure 5A). Cluster analysis using *K*-means clustering on the VIR-LTT panel identified four major clusters that varied in their allele frequencies (Figure 5B). These four clusters are represented on the PC plot where PC1 and PC2 explained 34.4 and 10.6% of the variability, respectively. Cluster 1 included 79 accessions mostly from Russia (84%) with other major groups of accessions originating from Azerbaijan (5%) and Armenia (2.5%). Cluster 2 included 29 accessions, mostly from Russia (28%), Tajikistan (10%), and Armenia (10%). Cluster 3 included 59 accessions mostly from Spain (17%), Ukraine (15%), and Russia (10%), and Cluster 4 included 100 accessions mostly from Russia (27%), Bulgaria (21%), and Ukraine (19%). The genomic additive relationship matrix confirmed the four clusters identified using *K*-means clustering (Supplementary Figure S2). Each cluster included accessions that are highly related to each other especially with respect to clusters 1, 3, and 4. Lower genomic additive relationships were observed among clusters compared to within clusters (Supplementary Figure S2). The average of the additive genetic relationship matrix within the CAP-LTT panel (0.14) was lower than within the VIR-LTT panel (1.15). The genomic additive relationship between both panels was very low at −0.46.

### Genome-Wide Association Study of LTT

**Figure 6:**
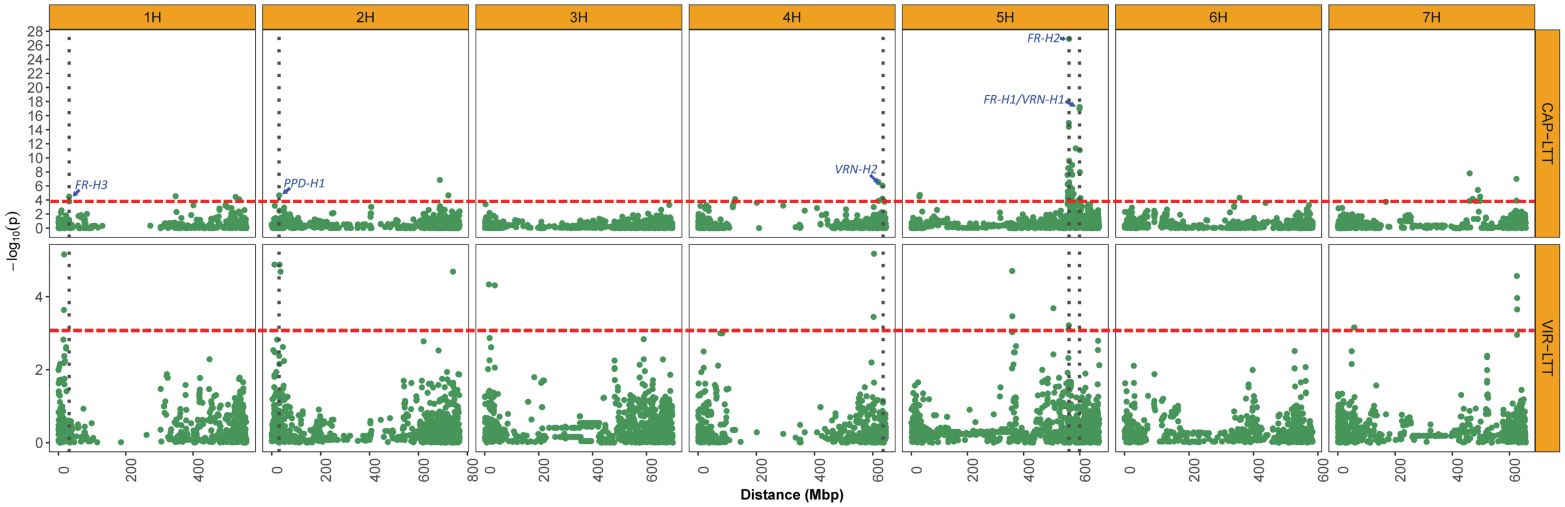
Manhattan plots displaying significant associations of WS for 882 accessions of the CAP-LTT panel and 267 accessions of the VIR-LTT panel. The thresholds for significant associations in both panels are shown as a red dashed horizontal line. Vertical dark gray dotted lines depict the positions of important WS genes that were identified in the CAP-LTT panel.

Genome-wide association studies was performed on the 267 VIR-LTT accessions using the Q + K model. A total of 12 MTAs for WS were identified in the panel and were positioned on every chromosome except 6H (Table 3; Figure 6). The names of MTAs, chromosomal and physical positions, marker names, values of *p* of detection, and gene annotations are summarized in Table 3. A genomic region on the short arm of chromosome 1H (VIR-MTA1) at 16.6 Mbp was associated with WS at the SNP marker SCRI_RS_150226 and explained 8% of the variability. The candidate gene for VIR-MTA1 is *HORVU1Hr1G007840* also located at 16.6 Mbp. On chromosome 2H, three MTAs were identified. One of these associations (VIR-MTA2) was linked to the SNP marker SCRI_RS_177330 at 11.3 Mbp on the short arm of 2H explaining 7% of the variability. The candidate gene for VIR-MTA2 is *HORVU2Hr1G004870* located at 11.0 Mbp on chromosome 2H. VIR-MTA3 also lies on the short arm of chromosome 2H and was positioned at 31.8 Mbp. It was identified by the SNP marker SCRI_RS_183655 and explained 7% of the variability. The candidate gene for VIR-MTA3 is *HORVU2Hr1G014550* also located at 31.8 Mbp on chromosome 2H. Given the low average adjacent marker LD (*r*^2^ = 0.17) in the 20 Mbp interval between VIR-MTA2 and VIR-MTA3, we conclude that these MTAs represent two distinct loci. VIR-MTA4 lies on the long arm of chromosome 2H linked to SCRI_RS_174071 at 740.9 Mbp and explained 6% of the variability for WS. The candidate gene for VIR-MTA4 is *HORVU2Hr1G118320* located within the haplotype at 742.2 Mbp on chromosome 2H (Table 3). Two MTAs were identified on the short arm of chromosome 3H: VIR-MTA5 was positioned at 15.3 Mbp based on its association with SNP marker SCRI_RS_97417, and VIR-MTA6 was positioned at 37.3 Mbp based on its association with SNP marker BOPA2_12_31298. Both of these MTAs explained 6% of the variability for WS. LD analysis revealed that the 22 Mbp genomic region harboring VIR-MTA5 and VIR-MTA6 is composed of several markers with low LD (*r^2^*<0.2) where the average adjacent marker LD was *r*^2^ = 0.29. The candidate gene for VIR-MTA6 is *HORVU3Hr1G016010* located within the haplotype at 38.3 Mbp on chromosome 3H. VIR-MTA7 was positioned on the long arm of chromosome 4H at 604.4 Mbp. It was linked to marker BOPA1_5245-304 and explained 9% of the variability for WS. The candidate gene for VIR-MTA7 is *HORVU4Hr1G077420* located at 604.0 Mbp on chromosome 4H (Table 3). Three MTAs were identified on chromosome 5H (Table 3). VIR-MTA8 was positioned at 358.8 Mbp through linkage with marker SCRI_RS_204494, whereas VIR-MTA9 and VIR-MTA10 lie at 505.7 and 560.6 Mbp through linkage with markers BOPA1_1896-1435 and BOPA2_12_30848, respectively (Table 3). The candidate genes for VIR-MTA8 and VIR-MTA9 are *HORVU5Hr1G046200* and *HORVU5Hr1G066250* located at 358.8 and 505.7 Mbp, respectively, on chromosome 5H (Table 3). *FR-H2*, located at 561.6 Mbp, is the candidate gene for VIR-MTA10 (Table 3; Muñoz-Amatriaín et al., 2020). Two MTAs were identified on chromosome 7H: VIR-MTA11 was positioned at 56.6 Mbp on the short arm through linkage with marker SCRI_RS_141629, and VIR-MTA12 was positioned at 625.8 Mbp on the long arm through association with marker SCRI_RS_93571. VIR-MTA11 and VIR-MTA12 explained 4 and 7% of the variation for WS. The candidate gene for VIR-MTA11 is *HORVU7Hr1G029770* located at 56.7 Mbp on chromosome 7H. Some of the candidate genes reported in the current study are known to play a role in providing cold stress tolerance (Table 3).

**Table 3:** Marker trait associations (MTAs) identified for winter survival in 267 VIR barley accessions grown in seven trials based on genome-wide association study.

MTA	Chromosome	Position (bp)^a1^	Markers (Variance explained)	*p*	Candidate gene^a2^	Gene position (Mbp)^a3^
VIR-MTA1	1H	16,644,372	SCRI_RS_150226 (0.08)	0.000007	*HORVU1Hr1G007840*	16.6
VIR-MTA2	2H	11,316,518	SCRI_RS_177330 (0.07)	0.000013	*HORVU2Hr1G004870*	11.0
VIR-MTA3	2H	31,772,845	SCRI_RS_183655 (0.07)	0.000013	*HORVU2Hr1G014550*	31.8
VIR-MTA4	2H	740,991,777	SCRI_RS_174071 (0.06)	0.000021	*HORVU2Hr1G118320*	742.2
VIR-MTA5	3H	15,255,602	SCRI_RS_97417 (0.06)	0.000046		
VIR-MTA6	3H	37,282,232	BOPA2_12_31298 (0.06)	0.000049	*HORVU3Hr1G016010*	38.3
VIR-MTA7	4H	604,403,849	BOPA1_5245-304 (0.09)	0.000007	*HORVU4Hr1G077420*	604.0
VIR-MTA8	5H	358,842,518	SCRI_RS_204494 (0.06)	0.000020	*HORVU5Hr1G046200*	358.8
VIR-MTA9	5H	505,672,865	BOPA1_1896-1435 (0.05)	0.000208	*HORVU5Hr1G066250*	505.7
VIR-MTA10	5H	560,570,415	BOPA2_12_30848 (0.05)	0.000607	*FR-H2*	561.6
VIR-MTA11	7H	56,624,480	SCRI_RS_141629 (0.04)	0.000696	*HORVU7Hr1G029770*	56.7
VIR-MTA12	7H	625,768,442	SCRI_RS_93571 (0.07)	0.000027		

a1 : Physical position of SNP markers (bp) using the barley genome assembly.

a2 : Candidate gene name is according to the barley genome annotation.

a3 : Physical position of the candidate genes in Mbp.Gene annotation for all significant associations is included. Variances explained by markers are included after marker names.

### Haplotype Analysis

**Figure 7:**
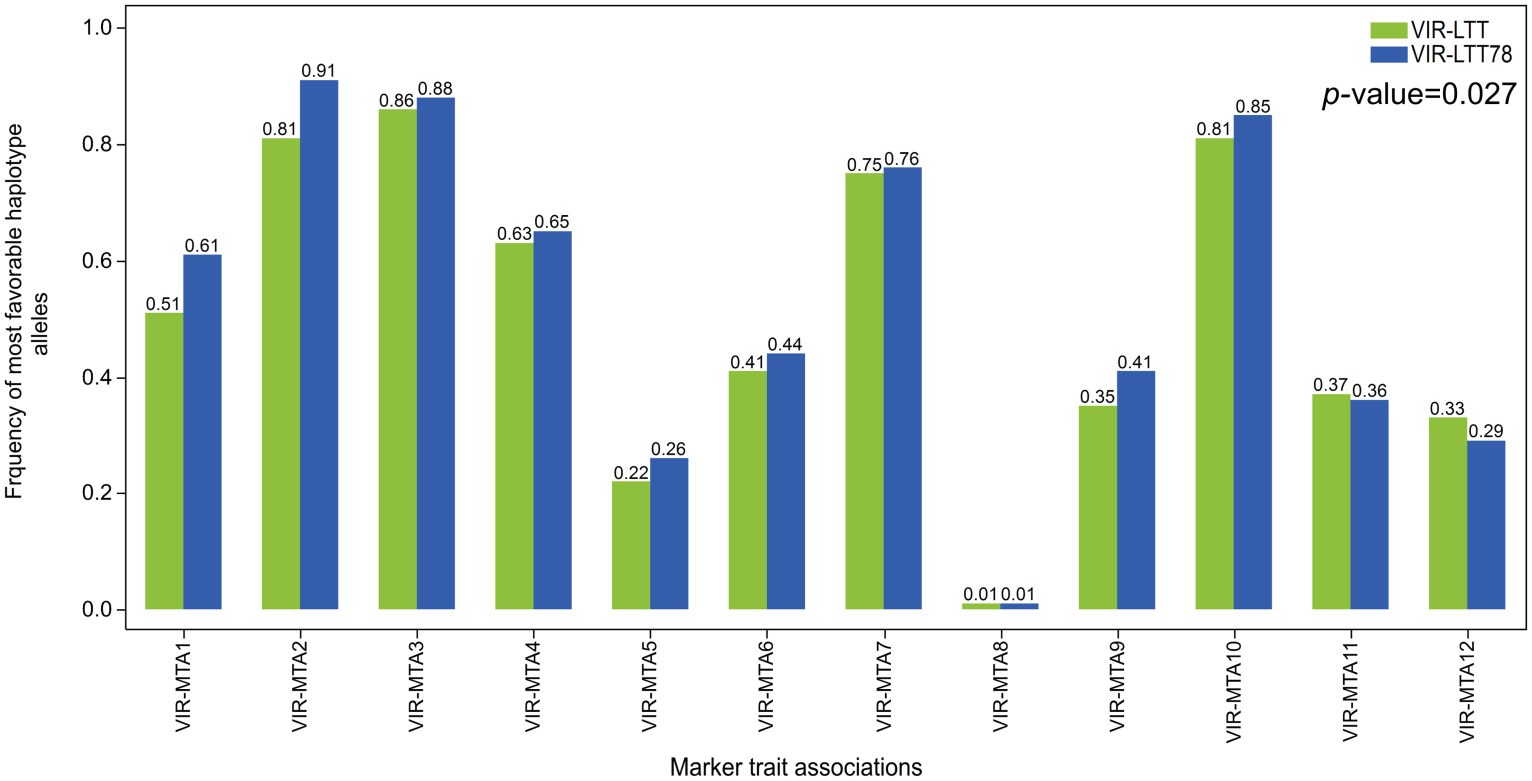
Frequency of the most favorable haplotype alleles based on genome-wide association study results for all MTAs in the VIR-LTT panel (all 267 accessions) and VIR-LTT78 subset (78 selected accessions).

Correction for differences in trial means was performed between the VIR-LTT and CAP-LTT panels using three common barley controls (Charles, Dicktoo, and McGregor) for accurate comparison of WS data between the two panels (Sallam and Smith, 2016). This was followed by calculation of the means across the 10 trials in the CAP-LTT panel. A fixed length for haplotype blocks of three adjacent markers was constructed in both the VIR-LTT and CAP-LTT panels. For haplotype analysis, each haplotype of three adjacent markers was considered a haplotype locus with several possible haplotype alleles (Yu and Schaid, 2007). The negative and positive values for the haplotype allelic scores indicate the reduced and improved allelic effects for WS, respectively. Within each panel, the haplotypes observed, expectation maximization estimates of haplotype frequencies, haplotype scores, and *p* values of associations for each haplotype are reported in Supplementary Tables S3 and S4. In the VIR-LTT panel, several haplotype alleles were observed for each of the 12 haplotypes (Supplementary Table S3). For example, VIR-MTA1 has six haplotype alleles with allele BBA (across the three markers SCRI_RS_88375, SCRI_RS_150226, and BOPA2_12_30241) occurring with a frequency of 49.6% and having the highest haplotype score of 4.23 (Supplementary Table S4). The number of haplotypes at the other MTAs were as follows: VIR-MTA2 (four alleles), VIR-MTA3 (four alleles), VIR-MTA4 (five alleles), VIR-MTA5 (five alleles), VIR-MTA6 (four alleles), VIR-MTA7 (six alleles), VIR-MTA8 (five alleles), VIR-MTA9 (six alleles), VIR-MTA10 (three alleles), VIR-MTA11 (three alleles), and VIR-MTA12 (six alleles). For the CAP-LTT panel, haplotype frequencies, haplotype scores, and association with WS were estimated for the previously identified 16 MTAs and ordered as previously reported by Muñoz-Amatriaín et al. (2020) (Supplementary Table S5). For these 16 MTAs, the number of haplotype alleles varied between 3 (CAPLTT-MTA12) and 8 (CAPLTT-MTA8; Supplementary Table S5). We examined the changes of haplotype allele frequencies for the most favorable alleles at the 12 significant MTAs between the VIR-LTT (all 267 accessions) and VIR-LTT78 (78 selected accessions; Figure 7). The VIR-LTT78 panel exhibited a significant overall increase in the most favorable haplotype alleles compared with the VIR-LTT panel (Figure 7). The greatest changes found in the most favorable haplotype allele frequencies for the VIR-LTT78 panel were with VIR-MTA1, VIR-MTA2, and VIR-MTA9. In other instances, the most favorable haplotype allele frequencies for the VIR-LTT78 panel remained unchanged or were lower than the VIR-LTT panel (Figure 7).

## Discussion

The VIR-LTT panel is a diverse and valuable germplasm resource for improving WS in barley. Climatic changes that increase temperature and prolong drought conditions can markedly affect crop production. To cope with climatic changes leading to increases in temperature in the northern hemisphere, autumn-sown crops can offer a solution by escaping high temperatures and drought conditions during the summer. This is particularly true for cool season annuals like barley. The development of facultative cultivars that can be sown in the spring or autumn is a major facet of the UM barley improvement program. The success of such cultivars when sown in autumn is largely dependent on their level of winter hardiness given the extremely low temperatures that often occur in the Upper Midwest region. Limited genetic diversity was a major challenge in efforts to improve WS in wheat (Fowler and Gusta, 1979). The introduction of germplasm from the Crimean region of the former USSR provided the critical genetic base for winter hardiness that facilitated the establishment of successful winter wheat breeding programs in the central Great Plains of the United States (Quisenberry and Reitz, 1974; Fowler and Gusta, 1979). To identify additional sources of winter hardiness for the UM barley improvement program, we evaluated a unique panel of barley germplasm from VIR in Minnesota during the severe winter of 2014–15. Remarkably, over 12% of the 2,214 accessions sown in the trial survived (i.e., the VIR-LTT panel) into the spring compared to less than 1% of Minnesota breeding lines in a parallel trial. Accessions in this VIR-LTT panel were surprisingly diverse with respect to their geographic origin, although the majority did originate from Russia and Ukraine (52%). The panel included landraces, breeding lines and cultivars; winter and facultative growth habit types; and both six-rowed and two-rowed spike types – the latter form present in just two of the 267 accessions.

Winter hardiness is challenging to improve through breeding due to the complexity of different environmental and genetic factors affecting the trait, foremost among them being low-temperature stress. To obtain the most robust data possible for genetic characterization and selection of winter hardiness in barley, it is important to evaluate the germplasm in multiple test sites and years to capture the range of different environmental conditions that will challenge the crop in the target regions. Even with multiple site testing, there will likely be extreme seasons where all or almost all lines die or alternatively survive the winter. In such cases, the selection intensity is too strong or too weak to be useful in breeding. The VIR-LTT accessions first selected in SP14.15 were evaluated in 10 additional environments to confirm their winter hardiness. The Minnesota locations, especially Crookston in the far northwest part of the state, represent the extreme for WS test sites, given the very low temperatures that frequently occur in winter. Indeed, in CK15.16 and CK16.17, temperatures dropped to −30°C for more than 10 consecutive days, and none of the accessions survived the winter. A similar result also occurred in SP16.17, although four VIR accessions survived. These three datasets were dropped from the final analysis due to no or very low survival. Other environments in Minnesota (SP15.16 and RS19.20); the additional northern tier states of Idaho (AB16.17), Montana (BZ16.17), Wisconsin (AL16.17), and New York (IT16.17); and the central Great Plains state of Nebraska (ME16.17) yielded useful data for assessing WS and its genetic architecture.

Based on the overall average and consistency for WS across trials, we selected the top 78 performing accessions from the VIR-LTT panel for additional testing in four environments (Figure 3A). The VIR-LTT78 subset of accessions displayed significantly (value of *p* < 0.000) higher WS across the four additional environments (84%) compared with the entire VIR-LTT panel across seven environments (68%) after correction for differences in the trial means using the two common barley controls of Charles and Dicktoo (Figure 3B). Several accessions from the VIR-LTT78 subset are being used as parents to improve WS in the UM barley improvement program. Within the VIR-LTT78 panel, 42 accessions have a facultative growth habit, a trait that is desirable for shortening the breeding cycle and providing flexibility for barley producers with respect to sowing time. WS for these 42 facultative accessions were slightly lower (82.0%) than the other 36 accessions with a winter growth habit (83.4%), but this difference was not statistically significant (*p* value 0.36; Supplementary Table S3). These results agree with a previous study demonstrating that facultative type barleys possess comparable levels of LTT and WS as winter type barleys (Rizza et al., 2011). The facultative barley cultivar Dicktoo was found to possess a high level of frost tolerance, similar to winter type barleys in a study that included 39 cultivars from North America and Europe (Karsai et al., 2001). After estimating the mean WS of accessions in the VIR-LTT78 panel across 11 trials, 64 performed better than Dicktoo (Supplementary Table S3). Of these 64 accessions, 35 were facultative, which clearly indicates the potential of this growth habit type for enhancing WS potential in barley breeding (Fernández-Calleja et al., 2021; Supplementary Table S3).

### The VIR-LTT Panel Is Genetically Diverse and Distinct From the CAP-LTT Panel

Heritability estimates for WS in barley range from moderate (0.36) to high (0.74; Rohde and Pulham, 1960). In the VIR-LTT panel, broad-sense heritability for WS was 0.46 across all seven environments, indicating that ample genetic variation for the trait is available in this germplasm. The VIR-LTT panel represents an excellent source of new alleles for enhanced winter hardiness that is genetically different from the CAP-LTT – the largest panel evaluated for winter hardiness to date (Figure 5A). The very low value (−0.46) for the average additive genetic relationship matrix between panels compared to within a panel (0.14 for the CAP-LTT and 1.51 for the VIR-LTT) indicates a high level of genetic differentiation between the panels.

One of the limitations of GWAS is the presence of subpopulations in the studied panel (Yu et al., 2006). Population stratification analysis identified four clusters in the VIR-LTT panel, with each cluster including accessions that are genetically related and from the same country of origin. Although PC1 and PC2 together explained a high percentage (45%) of the variability, there was no strong population stratification effect on WS as the means for cluster 1 (76.2%), cluster 2 (74.8%), cluster 3 (74.3%), and cluster 4 (73.5%) were very similar. These results indicate that while population structure does exist in the VIR-LTT panel, it did not have a strong impact on the average WS. However, it is possible that accessions within each cluster could contribute different combinations of favorable alleles for WS.

### Linkage Disequilibrium in the VIR-LTT Panel

The power of GWAS is dependent on the LD between markers and QTLs as estimated by *r*^2^ (Pritchard and Przeworski, 2001). The strength of LD between adjacent markers (*r*^2^ = 0.39) in the VIR-LTT panel was sufficient to capture most genetic variants and improve the power of QTL detection (Stich et al., 2007). Although LD was fairly uniform among chromosomes in the panel (*r*^2^ range of 0.36–0.43; Supplementary Figure S1), there was some variability in the pattern for specific chromosomes, especially around the middle of 1H and 4H. These two chromosomes also had the lowest number of markers (571 for 1H and 637 for 4H). However, the overall consistency of LD above *r*^2^ = 0.1 for more than 350 Mbp in the panel should be sufficient to detect significant QTLs, even for complex traits (Ersoz et al., 2009).

### Identification of Novel Winter Survival Associations and Candidate Gene Analysis

Genome-wide association studies for WS in the genetically diverse VIR-LTT panel identified 12 MTAs on all chromosomes except 6H (Table 3). Many of the MTAs identified were novel and associated with genes that could enhance WS in barley. However, some well-characterized genes known to contribute to winter hardiness in barley such as *FR-H1* and *FR-H3* were not identified in the VIR-LTT panel. This result may be due to the effect of allele frequency on QTL detection as the VIR-LTT panel contains the most winter hardy accessions from the original VIR panel of 2,214 accessions. Selection of the most winter hardy accessions from the original VIR panel may have altered allele frequencies (e.g., to near fixation for a specific allele), resulting in the inability to detect some WS QTLs as discussed below.

On chromosome 1H, the candidate gene for the novel VIR-MTA1 is *HORVU1Hr1G007840*, which encodes a cytochrome P450 superfamily protein (Table 3). These types of proteins play an important role in stress response as they are significantly upregulated during cold temperatures in both tall fescue (*Festuca arundinacea*) and perennial ryegrass (*Lolium perenne*; Pandian et al., 2020). Tao et al. (2017) analyzed the transcriptome of these two species and found that cytochrome P450 transcripts are responsive to cold stresses, providing evidence for the importance of cytochrome P450s in LTT. VIR-MTA1 lies about 14 Mbp upstream of *FR-H3* (Figure 6; Muñoz-Amatriaín et al., 2020), an important frost resistance gene. While physically close, the LD between the most significant markers at VIR-MTA1 and *FR-H3* was 0.01, indicating they are distinct loci. *HORVU1Hr1G012710* was identified as the candidate gene for *FR-H3* and encodes a lipid transfer protein induced by low temperature (Muñoz-Amatriaín et al., 2020). The novel VIR-MTA2 was highly associated with WS in the VIR-LTT panel, and the candidate gene at this locus (*HORVU2Hr1G004870*) encodes a NAD(P)-binding Rossmann-fold superfamily protein, which is involved in protein modification and signaling pathways (Zheng et al., 2007). The candidate gene at VIR-MTA3 (*HORVU2Hr1G014550*) encodes a protein WVD2-like 7 that regulates the orientation of microtubules. Interestingly, VIR-MTA3 was positioned on the short arm of chromosome 2H at 31.8 Mbp in close proximity to the photoperiod sensitivity gene *PPD-H1* at 29.9 Mbp (Muñoz-Amatriaín et al., 2020). While physically close, the LD between SCRI_RS_183655 at VIR-MTA3 and *PPD-H1* (BOPA2_12_10880) was 0.0, indicating they are distinct loci. *PPD-H1* was previously demonstrated to affect WS in barley (Muñoz-Amatriaín et al., 2020). Fowler et al. (2001) found that photoperiod sensitivity affected the expression of cold induced genes and consequently WS in the barley cultivar Dicktoo. Under short day conditions, these cold induced genes had greater expression. They also found that plants of Dicktoo grown under cold conditions and short days accumulate considerably more leaf biomass than plants grown under the same cold conditions and long days. This greater accumulation of leaf biomass may improve tolerance to cold conditions (Fowler et al., 2001). The candidate gene for VIR-MTA4 (*HORVU2Hr1G118320*) encodes phosphatidylinositol kinase proteins that were previously found to affect cold response in both maize (*Zea mays*) and Arabidopsis (*Arabidopsis thaliana*; Wang et al., 2016). VIR-MTA5 is a novel MTA for WS; however, no candidate gene was identified. The candidate gene for VIR-MTA6 (*HORVU3Hr1G016010*) encodes a SUMO (small ubiquitin-related modifier) that is involved in cold response (Johnson, 2004). A mutation in *SIZ1*, a SUMO E3 ligase signaling gene, resulted in freezing sensitivity in Arabidopsis due to a decrease in the expression of *CBF3*/*DREB1A*, a gene encoding transcription factors for acclimation to cold temperatures (Miura and Hasegawa, 2008). On chromosome 4H, the candidate gene for VIR-MTA7 is *HORVU4Hr1G077420*, which encodes a receptor kinase 2 leucine-rich repeat domain, instrumental to many biological processes in plants including development, response to pathogen attack and tolerance to abiotic stresses (Fischer et al., 2016). VIR-MTA8 was mapped at 358.8 Mbp on chromosome 5H. The candidate gene for VIR-MTA8 is *HORVU5Hr1G046200*, which lies at the same position. *HORVU5Hr1G046200* encodes a LURP1-related protein that was found to be involved in disease resistance in Arabidopsis (Baig, 2018) and is upregulated under cold conditions (Bittner et al., 2020). *HORVU5Hr1G066250* is the candidate gene for VIR-MTA9 on chromosome 5H and encodes a legumain-like cysteine protease (LLCP), also known as a vacuolar-processing enzyme. These type of enzymes are known to play a role in seed formation and germination, but more recently they were found to be regulated by cold stress in rice (Wang et al., 2018). This suggests a possible role of *HORVU5Hr1G066250* in cold stress tolerance. VIR-MTA10 on chromosome 5H was associated with WS based on marker BOPA2_12_30848. This marker is positioned 1 Mbp from SCRI_RS_23735, which was previously found significantly associated with WS by Muñoz-Amatriaín et al. (2020). Both markers are likely detecting the same MTA. A significant MTA was not found for SCRI_RS_23735 in the VIR-LTT panel. This was likely due to the fact that 265 of the 267 accessions (99.0%) carry the favorable allele at this marker, demonstrating the effect of strong selection for increasing the frequency of the desired marker allele to near fixation. The candidate gene for VIR-MTA10 is the frost resistance gene *FR-H2* (Figure 6), which encodes a C-repeat binding factor (CBF) that regulates cold acclimation and tolerance to freezing conditions (Knox et al., 2010). The candidate gene for VIR-MTA11 on chromosome 7H is *HORVU7Hr1G029770*, which encodes a FAD-dependent oxidoreductase family protein. Low-temperature conditions activate *FAD2* genes in plants, resulting in increased fatty acid content and consequently tolerance to cold conditions (Dar et al., 2017). VIR-MTA12 was also identified on chromosome 7H, but no candidate gene was identified. As summarized above, a number of candidate genes involved in response to cold temperatures were identified for WS MTAs in this study. This information will provide the basis for further molecular investigations of these genes and their contribution to LTT and winter hardiness.

### Haplotype Frequency, Effects, and Diversity in the VIR-LTT and CAP-LTT Panels

Genome-wide association studies panels, as opposed to bi-parental populations, are presumed to have more than two loci segregating at a locus. Therefore, it is useful to investigate multi-allelic haplotypes and their associations with causative variants. Haplotype loci that include polymorphic adjacent markers represent different combinations of alleles and can be more effective in capturing associations with the target traits compared with using single markers (Yu and Schaid, 2007). There are several advantages of using haplotypes associated with candidate genes including: (i) haplotypes are more biologically relevant than single markers because they are better able to capture high-order interactions, (ii) genetic variation in a population is determined by haplotypes which are ancestral and transmitted between generations, and (iii) greater power of association analysis is realized when using haplotypes compared to single markers (Clark, 2004; Liu et al., 2019). We estimated haplotype allele frequencies and haplotype scores to identify the favorable haplotype alleles for all significant MTAs in the VIR-LTT panel and the CAP-LTT panel. For *FR-H2*, the same marker haplotype (including: BOPA1_6170-304, SCRI_RS_237352, and BOPA2_12_30705) was present in both panels (Supplementary Tables S4 and S5). *FR-H2* was the only significant MTA that overlapped between both panels, and we confirmed that the most favorable haplotype allele at *FR-H2* (BAA) was the same in both panels across the three markers (Supplementary Tables S4 and S5). The favorable haplotype allele at *FR-H2* showed a significant association with WS in both panels, confirming the effectiveness of the haplotype analysis approach for selecting individuals carrying the favorable allele. This favorable haplotype allele occurred in a high frequency of 80.5 and 88.0% for the VIR-LTT and CAP-LTT panels, respectively (Supplementary Tables S4 and S5). The favorable haplotype allele at *FR-H2* was found in 70 accessions of the VIR-LTT78 subset (89.7%), indicating the importance of this gene in providing tolerance to cold conditions. The VIR-LTT panel is composed of 267 barley accessions selected from a larger VIR panel of 2,214 accessions after an initial WS experiment in SP14.15 that eliminated cold sensitive accessions. This may explain the higher frequency of favorable haplotype alleles (>50%) for six significant MTAs, including VIR-MTA2, VIR-MTA3, VIR-MTA4, VIR-MTA7, VIR-MTA8, and VIR-MTA10 (*FR-H2*; Supplementary Table S4). The *FR-H3* haplotype identified in the CAP-LTT panel was strongly associated with WS with the most favorable allele occurring at a frequency of 18.7%. Even though *FR-H3* was not identified in the VIR-LTT panel, 40 accessions in the VIR-LTT78 panel (51.3%) carried the favorable haplotype allele at the locus (data not shown). Within the VIR-LTT panel of 267 accessions, 126 (47.2%) carried the most favorable allele (BBB) at *FR-H3* across the three markers of BOPA2_12_30336, SCRI_RS_114047, and SCRI_RS_193392. The peak SNP marker for *FR-H3* in the CAP-LTT panel was not significant in the VIR-LTT panel. We cannot explain the reason why *FR-H3* was not identified using GWAS despite the high frequency of the favorable allele. However, comparison of haplotype alleles in both panels led to the identification of VIR-LTT accessions carrying the favorable allele at *FR-H3*. At *Vrn-H2* (CAPLTT-MTA9), two haplotypes were significantly associated with WS, and those haplotypes (AAA and AAB) occurred together at a frequency of 88.5% (Supplementary Table S5). *Vrn-H2* was not detected in the VIR-LTT panel; however, 143 accessions (53.6%) carried the favorable haplotype alleles. The *FR-H1/Vrn-H1* (CAPLTT-MTA12) favorable haplotype allele occurred at a frequency of 89% in the CAP-LTT panel and in the VIR-LTT panel was nearly fixed (98.5%), hindering the detection of this gene through GWAS. This result can be explained once again by the initial winter hardiness evaluation of the original VIR panel in SP14.15 that resulted in an increased frequency of favorable alleles for WS.

Using haplotype allelic scores, we characterized the number of favorable alleles in each accession of the VIR-LTT78 panel, an important analysis for parental selection in breeding. Fifteen accessions carried the most favorable haplotype alleles at 11 of the 12 MTAs identified in the current study including *FR-H2*. This list of accessions included VIR13976 and VIR13894, which both had WS rates of >83% across 11 trials. The majority of these 15 accessions are landraces originating from the Kabardino-Balkarian and Stavropol areas within the North Caucasus region of the Russian Federation. In addition to the favorable alleles at the 11 MTAs identified in the VIR-LTT panel, two accessions with winter growth habit (VIR13976 and VIR13894) also carried the favorable haplotype alleles at *FR-H3*, *Vrn-H*2, and *FR-H1*/*Vrn-H1*. Thus, the barley landraces selected and advanced through many generations in the North Caucasus region represent valuable sources of WS for breeding autumn-sown barley in the Upper Midwest.

We investigated the effects of selection for WS to increase the frequency of favorable haplotype alleles. This analysis was performed on 78 accessions having the highest and most consistent WS (i.e., the VIR-LTT78 panel) in comparison to the original VIR-LTT panel. We found that accessions in the VIR-LTT78 panel had a significantly higher frequency of the most favorable haplotype alleles compared with accessions in the VIR-LTT panel. WS is a complex trait with many contributing loci in addition to the previously described QTLs. In this study, we identified several accessions carrying a large number of favorable alleles, but no single accession carried all of the characterized favorable alleles at all loci associated with WS in both panels (data not shown). The goal of plant breeding is to increase the frequency of desired alleles beyond what is observed in the current germplasm. Therefore, breeding efforts should focus on combining all favorable WS alleles using haplotypes that encompass the significant associations identified from both the VIR-LTT and CAP-LTT panels.

Winter wheat and winter rye have comparatively higher levels of LTT than barley and are reliable crops in the cold-challenged regions of northwest Minnesota (i.e., Crookston), other northern tier states, and the southern Prairie Provinces of Canada. While the VIR-LTT panel suffered complete die-out in 2 years of field trials in northwest Minnesota, the survival rate of many accessions in south central Minnesota (i.e., St. Paul and Rosemount) was over 80%. Field trials conducted on autumn-sown barley in Minnesota indicate that winter survival rates of 80% or more do not result in significant yield reductions (Kevin Smith, unpublished). Thus, with a sustained breeding effort, it may be possible to develop autumn-sown facultative cultivars with a high level of winter hardiness enabling a new niche for barley cultivation in the region that will satisfy both industry needs for a local source of quality malting barley and public concerns about more ecologically friendly cropping systems.

In conclusion, the extensive phenotypic and genotypic data collected and analyzed from the VIR-LTT and CAP-LTT panels provides a valuable resource to greatly enhance breeding for increased WS in autumn-sown barley. The ability to improve WS in germplasm with a facultative growth habit without any apparent tradeoff should help accelerate breeding and provide flexibility to barley producers in changing environments. Based on the overall WS across environments, growth habits, row type, and haplotype scores, we identified several facultative and winter barley accessions from the VIR-LTT and CAP-LTT panels that could serve as ideal parents for a multiparent population with the purpose of accumulating favorable alleles for WS at the greatest possible number of loci.

## Data Availability Statement

The datasets presented in this study can be found in online repositories. The names of the repository/repositories and accession number(s) can be found in the article/Supplementary Material.

## Author Contributions

AS, BS, and KS contributed to the conception and design of the experiment and wrote the manuscript. IL and OK provided the germplasm for the study. AS, BS, TS, GH, JS, PB, JW, CD, ES, MS, and JE conducted the experiments and generated WS data. AS performed data analysis. All authors contributed to the article and approved the submitted version.

## Funding

This research was funded by the American Malting Barley Association (AMBA), Brewers Association, Lieberman-Okinow Endowment at the University of Minnesota, and Minnesota Agricultural Experiment Station Project No. MIN-22-085: Exploiting Wild Relatives for Cultivated Wheat and Barley Improvement.

## Conflict of Interest

The authors declare that the research was conducted in the absence of any commercial or financial relationships that could be construed as a potential conflict of interest.

## Publisher’s Note

All claims expressed in this article are solely those of the authors and do not necessarily represent those of their affiliated organizations, or those of the publisher, the editors and the reviewers. Any product that may be evaluated in this article, or claim that may be made by its manufacturer, is not guaranteed or endorsed by the publisher.

## Footnotes

^1^ https://galaxy-web.ipk-gatersleben.de/
