# Supplementary material for: Cold Conditioned: Discovery of Novel Alleles for Low-Temperature Tolerance in the Vavilov Barley Collection
Source: Front Plant Sci. 2021 Dec 15;12:800284. doi: 10.3389/fpls.2021.800284 (PMC8715003; doi:10.3389/fpls.2021.800284)

Figure S1: Heatmap for the distribution of linkage disequilibrium (LD) across the genome in the VIR-LTT panel estimated as r2 using 5,813 SNP markers. The number of SNP markers for each chromosome and average adjacent marker LD for each chromosome are included.

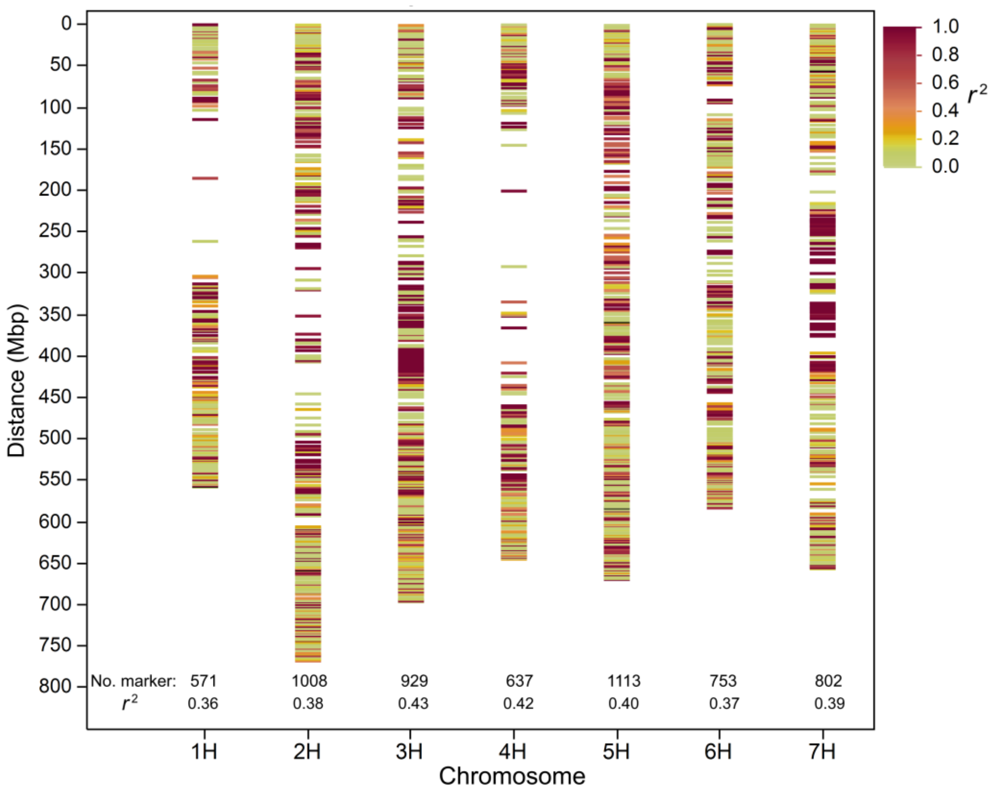

Figure S2: Heatmap matrix displaying the genomic additive relationship matrix for the 267 barley accessions of the VIR-LTT panel based on 5,813 SNP markers.

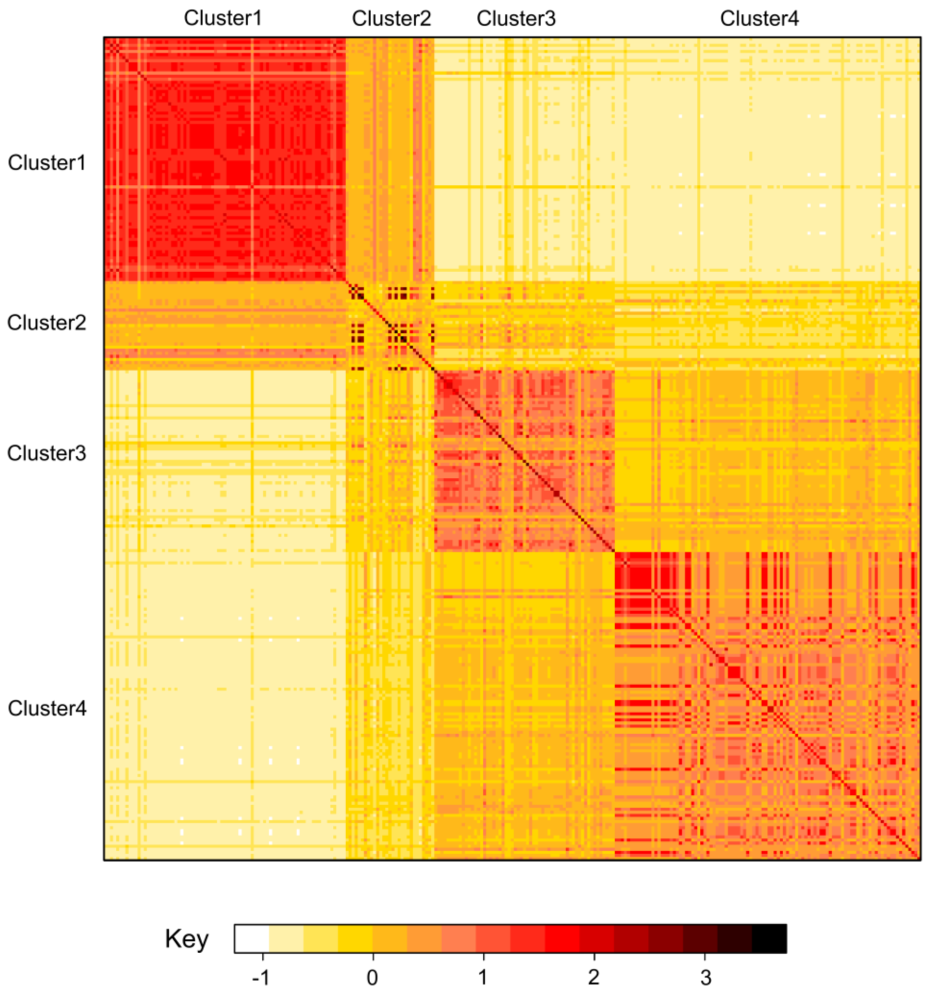

Supplement: Supplementary file 1 [file Data_Sheet_1.pdf]
